# The Effect of Concomitant Immunotherapy and Stereotactic Radiotherapy, and of Location on Survival in Patients With Brain Metastases From Melanoma

**DOI:** 10.1002/cam4.70923

**Published:** 2025-06-09

**Authors:** Chiel B. Nijboer, Djura Piersma, Angelique E. J. Sijben, Besim Hoti, Matthijs van der Meulen

**Affiliations:** ^1^ Department of Neurology Medisch Spectrum Twente Enschede the Netherlands; ^2^ Faculty of Medicine, Rijksuniversiteit Groningen Groningen the Netherlands; ^3^ Department of Internal Medicine Medisch Spectrum Twente Enschede the Netherlands; ^4^ Department of Neurology Leiden University Medical Center Leiden the Netherlands

**Keywords:** brain metastases, immunotherapy, melanoma, prognosis, radiation

## Abstract

**Background:**

Melanoma is one of the most common causes of brain metastases (BM). Despite recent therapeutic advances, survival in patients with brain metastases from melanoma (MBM) is dismal. In this study, we analyse the effect of concomitant treatment with stereotactic radiotherapy (SRT) and immunotherapy on survival, and of the location of BM on survival.

**Methods:**

All patients with MBM diagnosed in Medisch Spectrum Twente, between 2011 and 2023 were included. Patient, radiological, and treatment variables were retrospectively collected. The primary outcome was overall survival (OS) and the secondary outcome was intracranial progression‐free survival (IPFS). The effect of combination treatment (IRT), location, and other known prognostic factors on outcome measures was analysed using univariate and multivariate Cox regression. Location groups were divided into only supratentorial, only infratentorial, and both infra‐ and supratentorial BM.

**Results:**

92 patients with MBM were included. The mean age was 64 years with standard deviation (SD) 12, median OS [interquartile range (IQR)] was 9.8 months [4.0–31.0] in the total population. No difference in OS was found between different treatment regimens (*n* = 56). Patients having both infra‐ and supratentorial BM showed a significantly reduced OS compared to having only supratentorial BM in multivariate analysis (Hazard Ratio (HR): 2.81; 95% Confidence Interval (CI):1.37–5.74; *p* < 0.01). IRT had a significantly positive effect on IPFS in univariate analysis (HR: 0.32; 95% CI: 0.13–0.77; *p* = 0.01).

**Conclusion:**

Concomitant treatment of immunotherapy and SRT is associated with a prolonged IPFS compared to immunotherapy only. Having both infra‐ and supratentorial BM is an independent prognostic factor for a shorter OS.

## 1 Introduction

Melanoma is a malignant tumour that derives from epidermal melanocytes and is known to have a tendency to spread to the brain. Melanoma is the third most common solid cancer that leads to brain metastases (BM) following lung and breast cancer [1, 2, 3]. Among patients with melanoma, 10%–40% will develop brain metastases during the course of their disease [4, 5, 6, 7]. BM from melanoma are associated with a very poor prognosis and have a median overall survival (mOS) of less than 6 months [4, 5, 8]. The median survival of patients with MBM receiving systematic treatment and SRT is estimated to be 7.9 months [9].

Until 2011, the treatment options for brain metastases from melanoma (MBM) were radiation and neurosurgery (i.e., local therapies) or BRAF‐targeted therapy. The latter is only possible in patients with a BRAF V600 mutated melanoma. There is a new era with the introduction of immunotherapy: CTLA‐4 antibody targeting agents and PD‐1 and PDL‐1 inhibitors have been shown to be effective in the treatment of metastasized melanoma, including in patients with brain metastases from melanoma (MDM) [10]. Combining immunotherapy treatment with local therapies such as stereotactic radiotherapy (SRT) is hypothesised to have a synergetic effect in decreasing tumour growth [11]. A systematic review reported a beneficial effect of concomitant treatment on Overall Survival (OS) [9]. However, survival outcomes have a great variability among studies; therefore, the prognostic value of combining these treatments remains unclear [12, 13].

Separate from the effect of treatment on survival, there are a few studies that elucidated significant prognostic factors associated with worse survival outcomes: increased number of brain metastases, leptomeningeal involvement, presence of extracranial metastases, older age, absence of BRAF V600 mutation, and a low Karnofsky Performance Score (KPS) [4, 14]. While localization of BM determines the symptoms and is known to influence treatment options, to date, little is known about the prognostic role of location in OS.

The aim of this study is to analyse the prognostic effect of the concomitant treatment of immunotherapy and stereotactic radiotherapy on both OS and Intracranial Progression‐Free Survival (IPFS). In addition, we analysed the effect of the location of BM on OS and IPFS.

## 2 Methods

### 2.1 Data Collection

All patients with radiologically confirmed MBM, diagnosed between January 2011 and September 2023 at Medisch Spectrum Twente, Enschede, the Netherlands were included. Medisch Spectrum Twente is a large teaching hospital and one of the 14 melanoma cancer centers in the Netherlands. Patients were excluded when the first diagnostic MRI or treatment regimen was unavailable. Patient characteristics including age, sex, KPS and survival time were collected. KPS was either extracted from patients records and tumour board information or reconstructed by the authors (CBN and MvdM) based on patients' presenting symptoms at the time of diagnosis as documented in patient files. Tumour information, such as BRAF status, presentation (symptomatic/asymptomatic) and radiological characteristics, such as the number and location of the brain metastasis or metastases, location, leptomeningeal involvement and radiological response were also registered. All information was extracted from the electronic patient records and saved in a Castor database. Treatment characteristics were collected, including therapies before BM, treatment regimen after BM diagnosis, start and stop dates of therapies, and reason for initiating and stopping treatment.

### 2.2 Treatment Regimen Subdivision

Patients that received immunotherapy and/or stereotactic radiotherapy were categorised into three groups based on received treatment SRT only, immunotherapy only, and the IRT group (concomitant immunotherapy and SRT). The latter was defined as receiving SRT and immunotherapy within a time span of ≤ 9 weeks from each other, regardless of the sequence. This time span was based on previous studies [15, 16]. Also, an additional analysis of OS and IPFS was performed using patients that received concomitant treatment within 30 days, as this time span is also used in other studies [9]. Other treatments given, such as WBRT or targeted therapy before and after SRT and/or immunotherapy, were registered and compared between the three treatment groups. Patients with a performance status of KPS < 70 were excluded from analysis.

### 2.3 Radiological Subdivision

Only patients with an available T1 weighted Magnetic Resonance Imaging (MRI) with gadolinium contrast enhancement were included. Location of brain metastases in the brain parenchyma was divided into only supratentorial, only infratentorial, or both supratentorial and infratentorial, based on the first MRI on which MBM were diagnosed.

### 2.4 Statistical Analysis

Baseline characteristics were described with a mean and standard deviation (SD) when the data were normally distributed or median and interquartile range (IQR) for continuous variables and with numbers and proportions for discrete variables. Differences were analysed with an ANOVA when normally distributed and a Kruskal–Wallis test when not normally distributed. Differences between location, as well as treatment groups, were analysed using Chi‐square test or Fisher's exact test.

The survival analyses for treatment groups, as well as location groups, were done using a log‐rank test with a Kaplan Meier curve, and with univariate and multivariate Cox‐regression analyses [17]. Prognostic factors were considered independent when a threshold of *p* < 0.05 was reached.

OS was defined as the time from the diagnosis of BM until death from any cause. IPFS was defined as the time from the diagnosis of BM until progression of BM diagnosed on a follow‐up brain MRI, diagnosed by one experienced neuroradiologist. Patients who were lost to follow‐up or who were alive at the time of the last follow‐up (October 30, 2023) were censored at the date of their last follow‐up visit for OS analysis.

Patients without a follow‐up MRI scan showing progression were not included in the IPFS analysis. No multivariate analysis on IPFS could be performed due to small subgroups.

## 3 Results

### 3.1 Patient Characteristics

A total of 92 patients with MBM were included. The mean age at baseline of the total group was 64 years (SD = 12) and 57 (62.0%) patients were male. Forty‐one (44.6%) patients presented with a KPS of 90. The median Diagnosis‐Specific Graded Prognostic Assessment (DS‐GPA) was 2.0 [IQR 1–2.5] and the median follow‐up time was 9.3 months [IQR 3.5–21.5]. Thirty‐three patients (35.9%) had a BRAF V600 mutation. Seventeen patients (18.5%) had BM at the time of melanoma diagnosis, Table 1. After 1 year of follow‐up, the status of 95.7% (*N* = 88) was known.

**TABLE 1:** Patient and disease characteristics.

	*n*	%
Age in years (mean (SD))	64 (12)	
Gender		
Male	57	62.0
Female	35	38.0
KPS		
100	14	15.2
90	41	44.6
80	19	20.7
70	10	10.9
60	7	7.6
50	1	1.1
DS‐GPA [IQR]	2.0 [1–2.5]	
Overall survival in months [IQR]	9.8 [4.0–31.0]	
Follow‐up in months [IQR]	9.3 [3.5–21.5]	
BRAF V600 mutation	33	35.9
NRAS mutation	9	9.8
Other mutation	4	4.3
Unknown	5	5.4
Symptomatic presentation	66	71.7
Extracranial metastases	65	70.7
BM at diagnosis of melanoma	17	18.5
Time [IQR] between first diagnosis and presence BM (in months)	33.5 [6.8–90.3]	
Number [IQR] of BM	3 [1–9]	
Location		
Supratentorial	61	66.3
Infratentorial	5	5.4
Both	26	28.3
Leptomeningeal involvement	13	14.1
Targeted therapy (after BM diagnosis)	52	56.5
Resection	6	6.5
WBRT	19	20.7
No treatment/BSC	8	8.7
Treatment before diagnosis of BM		
Immunotherapy	14	15.8
Targeted therapy	9	9.8

*Note:* Clinical and demographical baseline characteristics of the study population at diagnosis of brain metastases (BM). Data are represented as proportions n with corresponding percentages, means with corresponding standard deviation (SD) or as medians with corresponding interquartile range (IQR).

Abbreviations: BM, Brain Metastasis; BSC, Best Supportive Care; DS‐GPA, Diagnosis Specific‐Graded Prognostic Assessment using Molecular Markers; KPS, Karnofsky Performance Score; WBRT, Whole Brain Radiation Therapy.

### 3.2 Treatment Groups Characteristics

Fifty‐six of the 92 patients received immunotherapy and/or stereotactic radiotherapy for BM. Fourteen patients (25%) received both immunotherapy and SRT, (i.e., IRT group), 35 (62.5%) patients received only immunotherapy, and the SRT group consisted of 7 patients (12.5%). In the IRT group, time between both treatments was ≤ 4 weeks in 10 patients, and 4–9 weeks in 4 patients. Four other patients that received both treatments were not included in this study group because the time between therapies amounted more than 9 weeks. One patient was excluded because of a KPS of < 70, the remaining patients received a myriad of other treatments and were not included in this analysis. In total, 56 patients were included in the analysis of treatment groups. Mean age, sex, and KPS were not significantly different between groups (data not shown). Extracranial metastases were more often present in the SRT group (*p* = 0.03). The SRT group showed a higher number of symptomatic lesions, with borderline significance (*p* = 0.06). No statistical significant differences were seen in location of BM.

In the IRT group, patients initially either received nivolumab + ipilimumab (*n* = 11), nivolumab monotherapy (*n* = 1) or ipilimumab monotherapy (*n* = 2). In the immunotherapy group, most patients received nivolumab + ipilimumab (*n* = 24). Nivolumab monotherapy (*n* = 5), ipilimumab monotherapy (*n* = 4), and pembroluzimab (*n* = 2) were given in fewer cases. In the SRT group, three patients underwent surgery for BM, which is significantly more often compared to the other groups (*p* < 0.01). For other variables, no significant statistical differences were observed.

### 3.3 Location Groups Characteristics

Sixty‐one/92 patients had only supratentorial BM, and five patients had only infratentorial BM. The group that had both infra‐ and supratentorial lesions consisted of 26 patients. No significant difference in mean age, KPS, or sex was observed. Patients with both infra‐ and supratentorial lesions had a statistically significant higher number of BM (*p* < 0.01) than patients with only infratentorial or only supratentorial BM. Also, leptomeningeal involvement was more often present in the groups that had both infra‐ and supratentorial lesions, although this difference was not statistically significant (*p* = 0.10). BRAF V600 mutations were noticeably more present in the supratentorial only group, but this difference was not significant (*p* = 0.13).

### 3.4 Overall Survival

#### 3.4.1 Treatment

**FIGURE 1:**
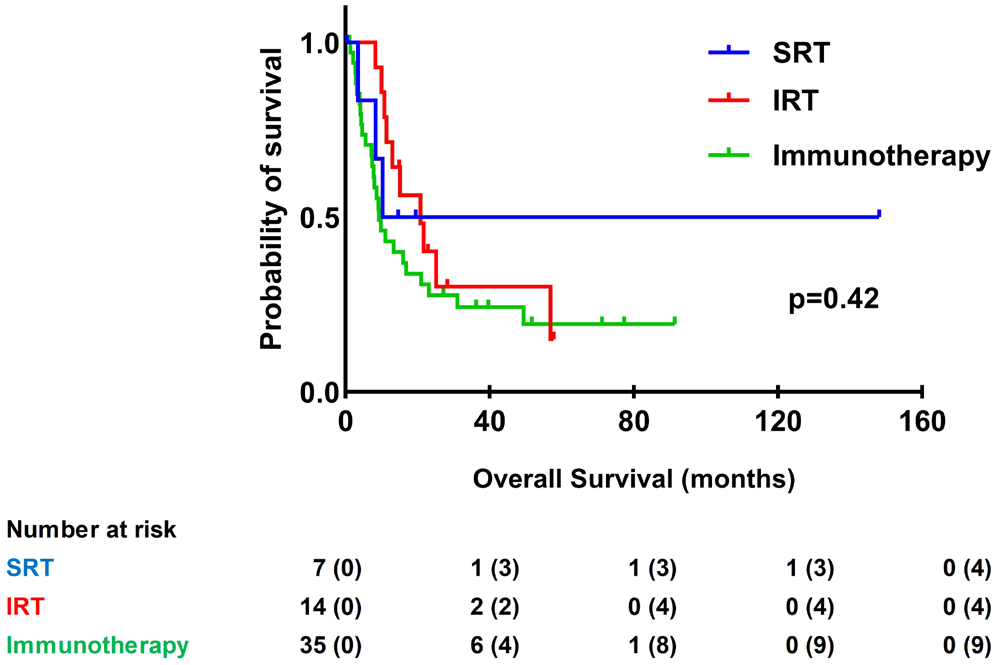
Kaplan–Meier curve for OS in months for different treatment groups. IRT, Concomitant immunotherapy and SRT (*n* = 56); SRT, Stereotactic radiotherapy.

No significant difference in OS between treatment groups (log‐rank test, *p* = 0.42) was observed. In treatment subgroups, a total of 39/56 (69.6%) patients died. The IRT group had a mOS of 20.8 months [IQR 11.5–56.9]. In those treated with SRT only and those with immunotherapy only, the mOS was 10.3 months [IQR 8.4–19.6] and 9.3 months [IQR 4.6–31.0] respectively, Figure 1.

Another analysis was performed for OS using only patients that received concomitant treatment within 30 days in the IRT‐group, in which no significant difference was found (log‐rank test, *p* = 0.53), Figure S4.

#### 3.4.2 Location

**FIGURE 2:**
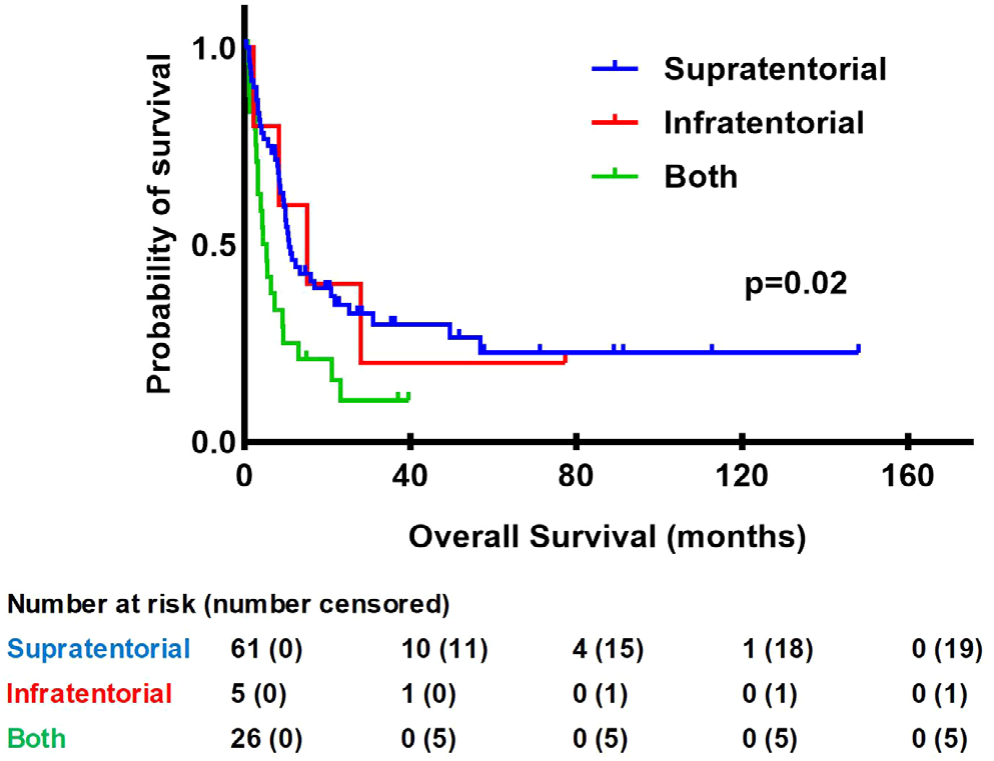
Kaplan–Meier curve for OS in months for different location groups (*n* = 92).

In the analysis of the effect of location, all 92 patients were included. A significant difference (log‐rank test, *p* = 0.02) was seen between the three different location groups, in which patients with MBM both supra‐ and infratentorial had the worst prognosis, Figure 2. In the location subgroups, a total of 67 (72.8%) patients died during follow‐up. The groups that had both infra‐ and supratentorial lesions had a mOS of 5.25 months [IQR 3.0–13.0]. In those patients with only supratentorial BM, and those who had only infratentorial BM, the mOS was 10.8 months [IQR 5.7–56.9] and 15.1 [IQR 8.3–28.0], respectively. A significant difference between only supratentorial BM and both infra‐ and supratentorial BM was found (*p* < 0.01), Figure S1. No significant difference in OS between the other location subgroups was found, Figures S2 and S3.

#### 3.4.3 Cox Regression Analyses for OS

In univariate Cox regression, having a higher KPS at baseline was associated with a better OS (HR: 0.70; 95% CI: 0.55–0.88; *p* < 0.01). In univariate analysis only, 5–9 BM and > 9 BM were associated with a significantly worse prognosis compared to the group having 1–4 BM. Targeted therapy before diagnosis of BM was associated with a significantly worse OS in multivariate analysis (HR: 3.19; 95% CI: 1.32–7.71; *p* = 0.01). When started after diagnosis of BM, targeted therapy was strongly associated with a significantly better OS (HR: 0.48; 95% CI: 0.28–0.84; *p* = 0.01). The univariate Cox regression analyses showed a significantly worse OS in patients with both supra‐ and infratentorial lesions when compared to only supratentorial lesions, Table 2.

**TABLE 2:** Uni‐ and multivariate cox regression analyses of overall survival.

Variable	Univariate analysis		*p*	Multivariate analysis		*p*
	HR	95% CI		HR	95% CI	
Patient characteristics						
Age	1.02	0.99–1.04	0.16			
Gender						
Female	1.26	0.76–2.09	0.38			
KPS (factor 10)	0.70	0.55–0.88	< 0.01	0.64	0.50–0.83	< 0.01
Tumour characteristics						
BRAF V600	0.67	0.41–1.09	0.10			
Symptomatic presentation	0.90	0.53–1.51	0.67			
Extracranial metastases	1.58	0.91–2.75	0.11	2.02	1.10–3.71	0.02
Radiological characteristics						
Number of BM						
1–4	Ref			Ref		
5–9	2.12	1.14–3.96	0.02	0.90	0.39–2.10	0.81
> 9	2.24	1.24–4.03	< 0.01	2.24	1.09–4.63	0.03
Location						
Supratentorial	Ref			Ref		
Infratentorial	1.02	0.37–2.84	0.97	1.43	0.50–4.10	0.50
Both	2.10	1.23–3.57	< 0.01	2.81	1.37–5.74	< 0.01
Leptomeningeal involvement	1.76	0.92–3.37	0.09			
Treatment characteristics						
Treatment subgroups						
Immunotherapy + SRT (≤ 9 weeks)	0.66	0.32–1.37	0.26			
Immunotherapy	Ref					
SRT	0.60	0.18–1.98	0.40			
Targeted therapy (after BM diagnosis)	0.68	0.42–1.10	0.12	0.48	0.28–0.84	0.01
Resection of BM	0.04	< 0.01–1.19	0.06			
Treatment before diagnosis of BM						
Targeted therapy	2.29	1.08–4.86	0.03	3.19	1.32–7.71	0.01
Immunotherapy	1.60	0.83–3.07	0.16			

Abbreviations: BM, Brain Metastases; CI, confidence interval; HR, Hazard Ratio; KPS, Karnofsky Performance Score; ref., reference; SRT, Stereotactic Radiotherapy.

When corrected for KPS, extracranial progression, number of BM, targeted therapy before and after BM, having both infra‐ and supratentorial metastases remained statistically significant, Table 2.

### 3.5 Intracranial Progression‐Free Survival

**FIGURE 3:**
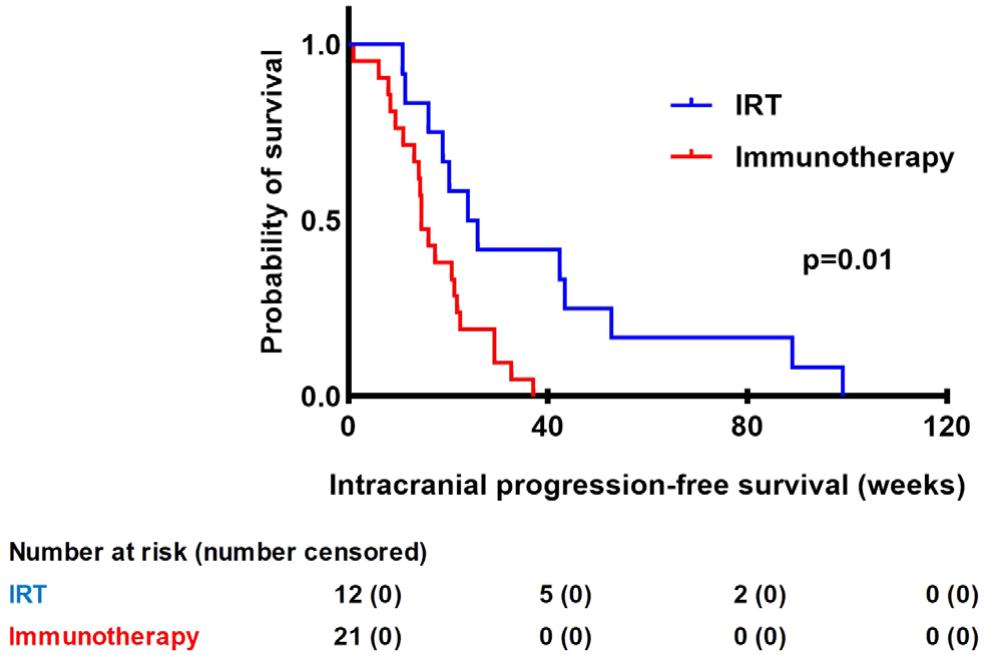
Kaplan Meier curve of intracranial progression‐free survival in weeks for different treatment groups. No patients were censored. IRT, Immunotherapy and stereotactic radiotherapy.

In the IRT group, the IPFS was significantly better with a median IPFS of 25.0 weeks [IQR 17.5–48.1], compared to those who received immunotherapy only: median IPFS 14.6 weeks [IQR 11.0–21.9], *p* < 0.01, Figure 3. The IRT group consists of 12 patients, while the immunotherapy only group consists of 21 patients. The SRT only group was not included in this analysis because only 2 patients had the event; therefore, no statistical power could be achieved.

Also for IPFS, another analysis was performed using only patients that received concomitant treatment within 30 days, which revealed a similar significance (log‐rank test, *p* < 0.01), Figure S5.

#### 3.5.1 Cox Regression Analysis for IPFS

The impact of variables on IPFS was tested in univariate Cox regression analysis. In this analysis, concomitant treatment of immunotherapy and stereotactic radiotherapy (IRT) was the strongest predictor of a prolonged IPFS (HR: 0.32; 95% CI: 0.13–0.77; *p* = 0.01). Targeted therapy before the presence of BM showed a trend towards significantly worse IPFS in univariate analysis but had a very wide confidence interval (HR 3.15; 95% CI: 0.81–15.23; *p* = 0.09). Increasing age showed a trend towards a significantly shorter IPFS (*p* = 0.08). The association between the location of BM and IPFS was not found. Leptomeningeal involvement showed a non‐significant trend towards a worse IPFS (*p* = 0.10). Univariate analysis is shown in Table 3.

**TABLE 3:** Univariate cox regression analyses of intracranial progression‐free survival.

Variable	Univariate analysis		*p*
	HR	95% CI	
Patient characteristics			
Age	1.03	0.99–1.05	0.08
Gender			
Female	1.08	0.57–2.03	0.81
KPS (factor 10)	1.01	0.97–1.05	0.56
Tumour characteristics			
BRAF V600	1.06	0.55–2.04	0.87
Symptomatic presentation	0.45	0.22–0.90	0.02
Extracranial metastases	1.63	0.86–3.12	0.14
Radiological characteristics			
Number of BM	1.02	0.98–1.07	0.34
1–4	Ref		
5–9	1.0	0.48–2.11	1.0
> 9	1.23	0.55–2.77	0.61
Location			
Supratentorial	Ref		
Infratentorial	1.57	0.21–11.85	0.66
Both	1.44	0.75–2.78	0.27
Leptomeningeal involvement	2.81	0.84–9.35	0.09
Treatment characteristics			
Treatment subgroups			
Immunotherapy + SRT	0.32	0.13–0.77	< 0.01
Immunotherapy	Ref		
SRT			
Targeted therapy (after BM diagnosis)	0.76	0.37–1.55	0.45
Resection of BM			
Treatment before diagnosis of BM			
Targeted therapy	3.51	0.81–15.23	0.09
Immunotherapy	0.90	0.32–2.53	0.84

Abbreviations: BM, Brain Metastasis; CI, confidence interval; HR, Hazard Ratio; KPS, Karnofsky Performance Score; SRT, Stereotactic radiotherapy.

## 4 Discussion

The aim of this study was to analyse both the impact of location and the impact of concomitant immunotherapy and stereotactic radiotherapy on both overall survival and intracranial progression‐free survival in patients with brain metastases from a melanoma.

In our study of 56/92 patients with MBM received immunotherapy and/or stereotactic radiotherapy. We found that combining immunotherapy and SRT does not significantly influence OS. Our finding of no difference in OS contradicts current literature that does point towards a synergetic effect of combining these treatments [15, 18]. Possible explanations for our finding could be the small sample size of patients receiving both treatments in our study (*n* = 14/56) and a relatively greater portion of patients in the IRT group (21.4%) receiving immunotherapy before the presence of BM when compared to immunotherapy only (5.7%). However, according to our Cox‐regression analysis, having had immunotherapy before the diagnosis of BM did not influence prognosis.

The median OS varied among subgroups. The IRT group, receiving both immunotherapy and stereotactic radiotherapy (SRT), had the longest median OS at 20.8 months [IQR 11.5–56.9]. In contrast, patients treated with SRT alone and those receiving only immunotherapy had shorter median OS: 10.3 months [IQR 8.4–19.6] and 9.3 months [IQR 4.6–31.0], respectively. Although these differences were not statistically significant, they suggest a potential benefit of combined therapy.

There was, however, a significant benefit of combination therapy in prolonging IPFS, with a median of 25.0 weeks [IQR 17.5–48.1] in the IRT group compared to 14.6 weeks [IQR 11.0–21.9] for the immunotherapy only group (*p* < 0.01). The effect on IPFS is a promising result and shows that concomitant treatment may decrease the intracranial progression rate compared to immunotherapy alone. Of all tested variables, concomitant treatment with immunotherapy and SRT (IRT) was the strongest predictor of prolonged IPFS. Interestingly, symptomatic presentation was associated with a lower risk of progression, which may reflect a selection bias where symptomatic patients receive more aggressive or earlier intervention. The beneficial effect of concomitant treatment with stereotactic radiotherapy and immunotherapy may be explained by several mechanisms. Firstly, stereotactic radiotherapy increases the permeability of the blood–brain barrier, allowing better penetration of immunotherapeutic agents into the brain parenchyma. Secondly, radiation induces mitotic cell death within the irradiated lesions, leading to the release of tumour antigens. These antigens can then stimulate a cytotoxic immune response and activate immune cells to recognise and attack tumour cells both within and outside the irradiated zone [11, 19]. This synergistic interaction between SRT and immunotherapy enhances the anti‐tumour immune response, potentially contributing to improved IPFS observed in our study.

We chose a timespan of ≤ 9 weeks in which SRT and immunotherapy were given, in line with a previous study [15]. Moreover, it was chosen based on clinical pharmacokinetics and data on treatment failure rates following SRT, suggesting it provides an optimal window for a synergistic effect. Data on immunotherapy pharmacokinetics indicate that most patients reach serum steady state levels between 9 and 18 weeks after beginning treatment [16]. The low rate of treatment failure within 9 weeks post‐SRT implies a sustained therapeutic effect during this period [20]. In our study, additional analyses were conducted, including patients having an interval of ≤ 4 weeks between treatments. However, no significant differences in outcomes were observed in this analysis, likely due to the relatively large proportion of patients that had already received both treatments within 4 weeks (*n* = 10/14) (Figure S4). Murphy et al. also showed an improved intracranial progression‐free survival in patients receiving immunotherapy and stereotactic radiotherapy, using a time interval of 4 weeks between treatments [21].

The effect of location, analysed in all 92 included patients, significantly impacted OS (log‐rank test, *p* = 0.02). Patients with both supra‐ and infratentorial BM had the worst prognosis with a mOS of 5.25 months. In multivariate analysis, after correcting for KPS, number of BM, targeted therapy before, and targeted therapy after presence of BM, this significance remained. Thus, localization in both tentorial regions is an independent prognostic factor. The significant difference between only supratentorial and both supra‐ and infratentorial localization is in line with two similar studies that defined the same subgroups, analysing the role of location specifically in BM patients from a melanoma: Staudt et al. (*n* = 265) studied prognostic factors in metastasized melanoma and reported a significantly shorter OS in patients having lesions in both supra‐ and infratentorial regions in univariate analysis [8]. Meier et al. (*n* = 100) also studied factors influencing survival and found that having only supratentorial BM is associated with a better OS compared to having BM in both infra‐ and supratentorial regions [22]. In our study, there was no significant difference in OS between having only supratentorial and only infratentorial BM. No effect of location on IPFS was seen in this study. This result could not be compared as other studies did not use IPFS as an endpoint.

In addition to the above mentioned findings, we found a few other prognostic factors for OS. First, the strongest predictor of an increased OS was a high KPS at baseline. Second, having 5–9 and > 9 BM was associated with a significantly worse outcome in univariate analysis, compared to the group having 1–4 BM. This significance lasted in multivariate analysis only for the category of > 9 BM. These findings are in line with current prognostic models, and a study done by Bander et al., in which having ≥ 5 BM was associated with a worse survival [23, 24]. Furthermore, targeted therapy after diagnosis of BM was associated with a prolonged OS. Targeted therapy before the presence of BM, however, was associated with a decreased OS. This can be explained by the window in which targeted therapy is effective and the decreased effectiveness over time. In patients having a high tumour load, targeted therapy is often started early in the disease course. In other words, when targeted therapy is started before the presence of BM, it is no longer as effective after BM diagnosis, therefore ultimately compromising chances of survival after this stage.

Besides that, age had no effect on OS in our study. We used a continuous variable for age to reflect biological reality. This approach may explain why age did not significantly influence the outcomes in our study.

In our study, KPS did not a significantly impact IPFS, which may be attributed to the small size of the subgroups analysed for IPFS (IRT: *n* = 12/92, Immunotherapy: *n* = 21/92). This limited sample size resulted in almost all individuals included in the IPFS analysis having a KPS of 90, reducing the variability needed to detect any potential impact related to KPS. In this cohort, only 30% of patients had a BRAF V600 mutation, which represents the higher age of our cohort, since in the elderly there is a lower percentage of BRAF V600 mutations in melanoma [25]; in the total population, approximately 50% of MBM patients have a BRAF V600 mutation [24].

The strengths of this study are that we analysed the effect of both a prognostic radiological factor (i.e., location) and the effect of different treatment modalities specifically for brain metastases in melanoma in a relatively large population (*n* = 92). To our knowledge, this is the first study that was able to identify location as an independent prognostic factor corrected for aforementioned important prognostic factors (such as number of BM) in multivariate analysis. Although this study was retrospective, there was a limited amount of missing data, and the dataset was rather comprehensive. However, the study has some limitations. First, follow‐up imaging data on extracranial progression is missing in our current database, and therefore, analysis on the progression‐free survival due to systemic progression could not be carried out. Instead, an analysis of IPFS was done, hampered; however, due to the lack of MRI that confirmed brain progression. Furthermore, some subgroups became very small, leading to smaller statistical power. We found an effect of the location on OS; however, we did not take into account the diameter or volume of BM. It is possible that the latter factors are of more prognostic value than location alone [26, 27].

In conclusion, this study shows that concomitant treatment regimen with immunotherapy and SRT is strongly associated with a prolonged IPFS. In addition, having both infra‐ and supratentorial BM is an independent prognostic factor for a worse OS.

## Author Contributions

**Chiel B. Nijboer:** conceptualization (lead), data curation (lead), formal analysis (lead), investigation (lead), methodology (equal), project administration (equal), visualization (lead), writing – original draft (lead). **Djura Piersma:** writing – review and editing (supporting). **Besim Hoti:** data curation (supporting), methodology (supporting), writing – review and editing (supporting). **Angelique E. J. Sijben:** writing – review and editing (supporting). **Matthijs van der Meulen:** investigation (lead), methodology (equal), project administration (lead), supervision (lead), validation (lead), writing – review and editing (lead).

## Ethics Statement

The study was approved by the institutional review board of Medisch Spectrum Twente (MST), number K21‐05. Patient consent was waived by the IRB due to the retrospective and non‐invasive nature of this study.

## Conflicts of Interest

The authors declare no conflicts of interest.

## Supporting information


Data S1.

## Data Availability

The data that support the findings of this study are available on request from the corresponding author. The data are not publicly available due to privacy or ethical restrictions.
