# Supplementary material for: The Effect of Concomitant Immunotherapy and Stereotactic Radiotherapy, and of Location on Survival in Patients With Brain Metastases From Melanoma
Source: Cancer Med. 2025 Jun 9;14(11):e70923. doi: 10.1002/cam4.70923 (PMC12146586; doi:10.1002/cam4.70923)
Supplement: Supplementary file 1 — Data S1. [file CAM4-14-e70923-s001.docx]

**Supplemental
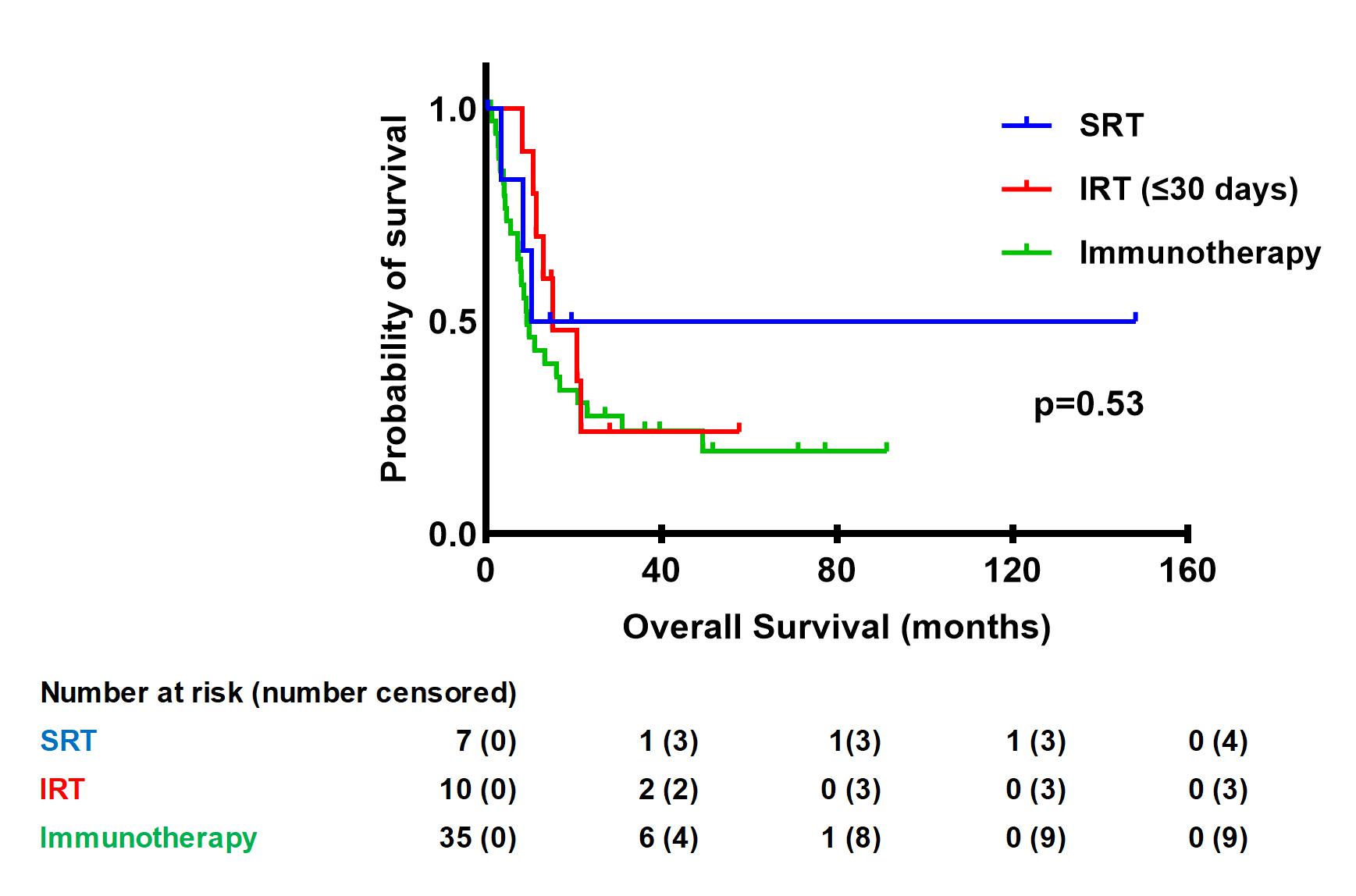

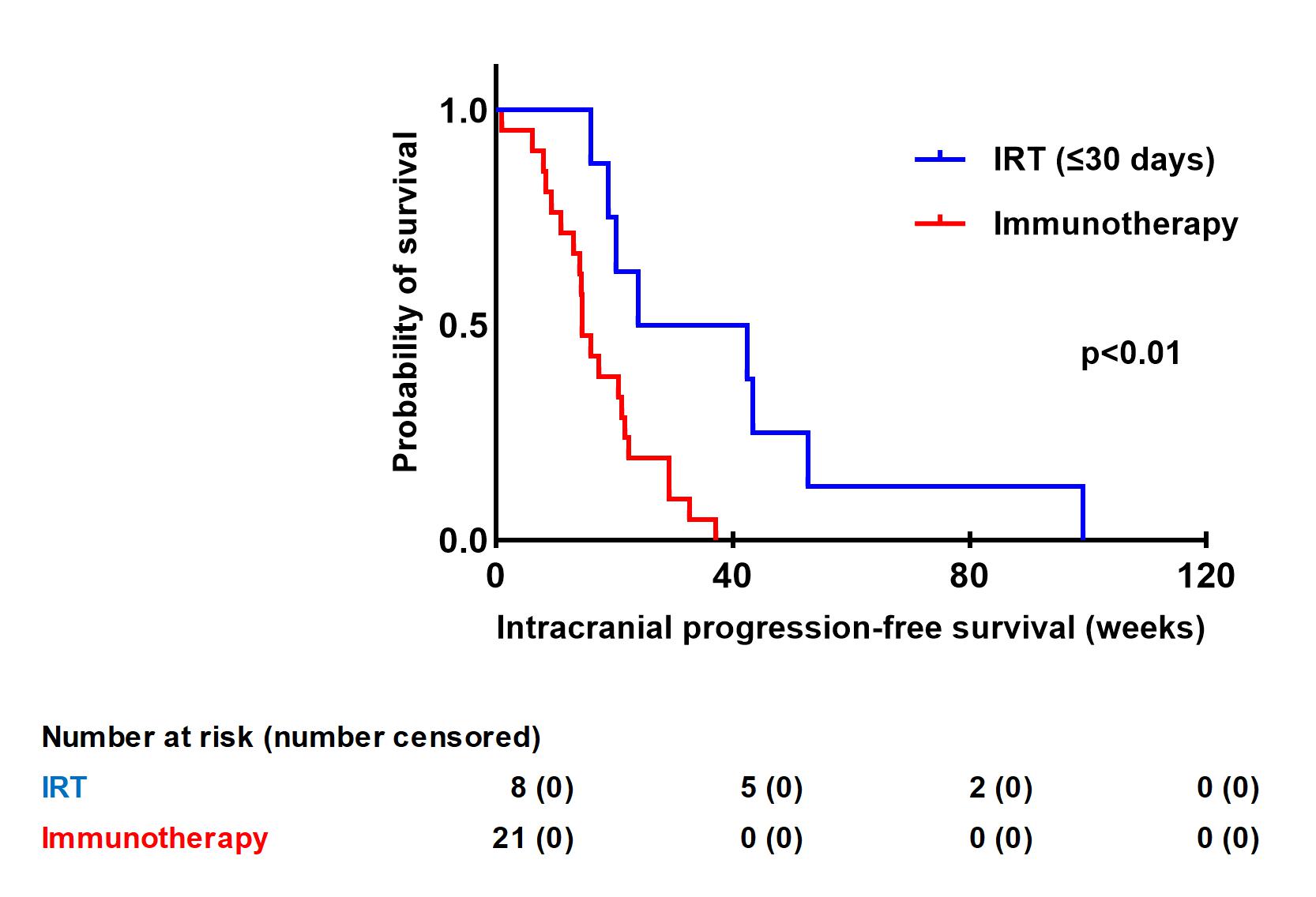
**

Supplemental Figure 1: Kaplan Meier curve for OS in months for different treatment groups. SRT: stereotactic radiotherapy. IRT: concomitant immunotherapy and SRT.

Supplemental Figure 2: Kaplan Meier curve for IPFS in weeks for different treatment groups, using patients that received concomitant treatment within 30 days for the IRT group. IRT: concomitant immunotherapy.

**
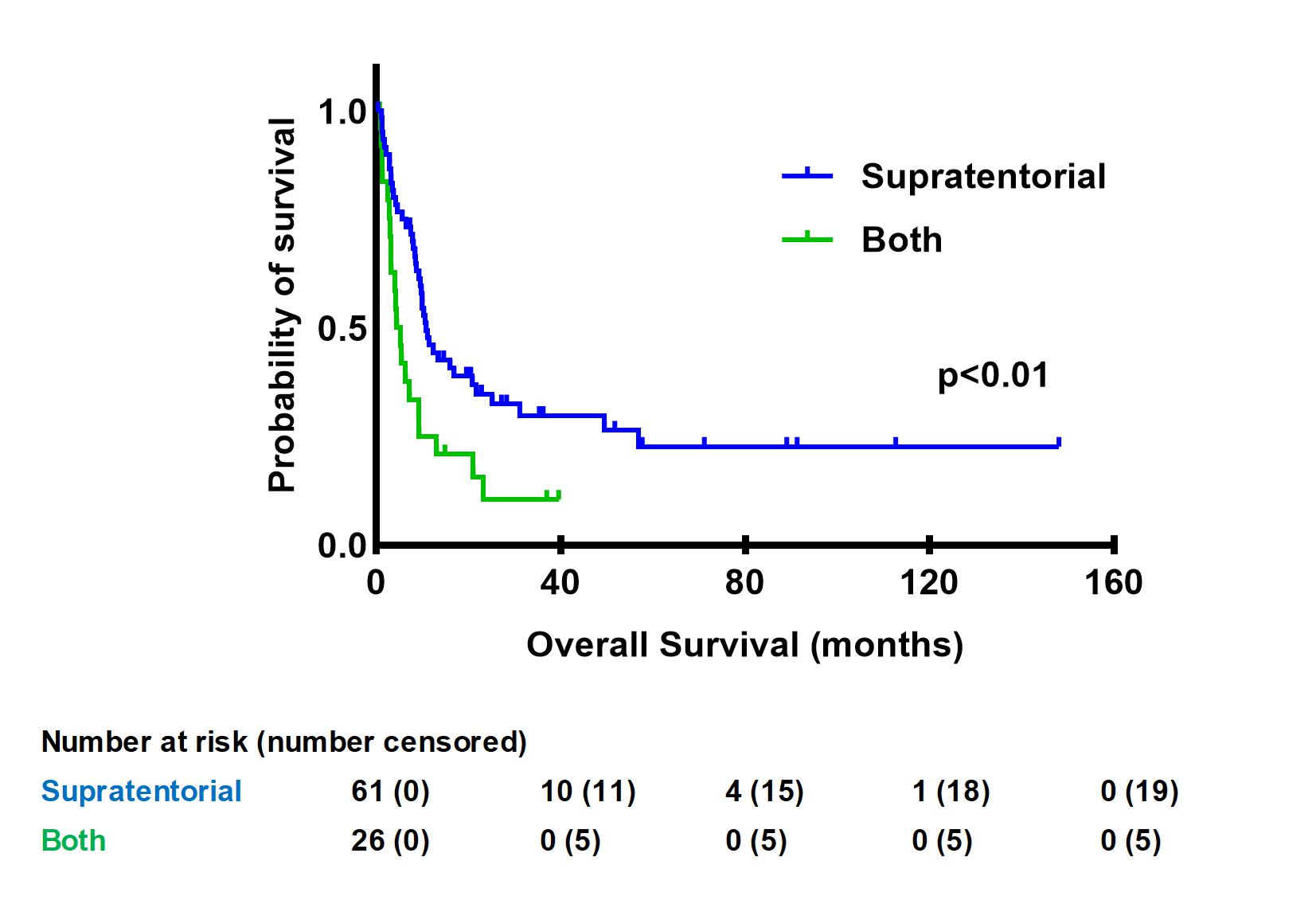

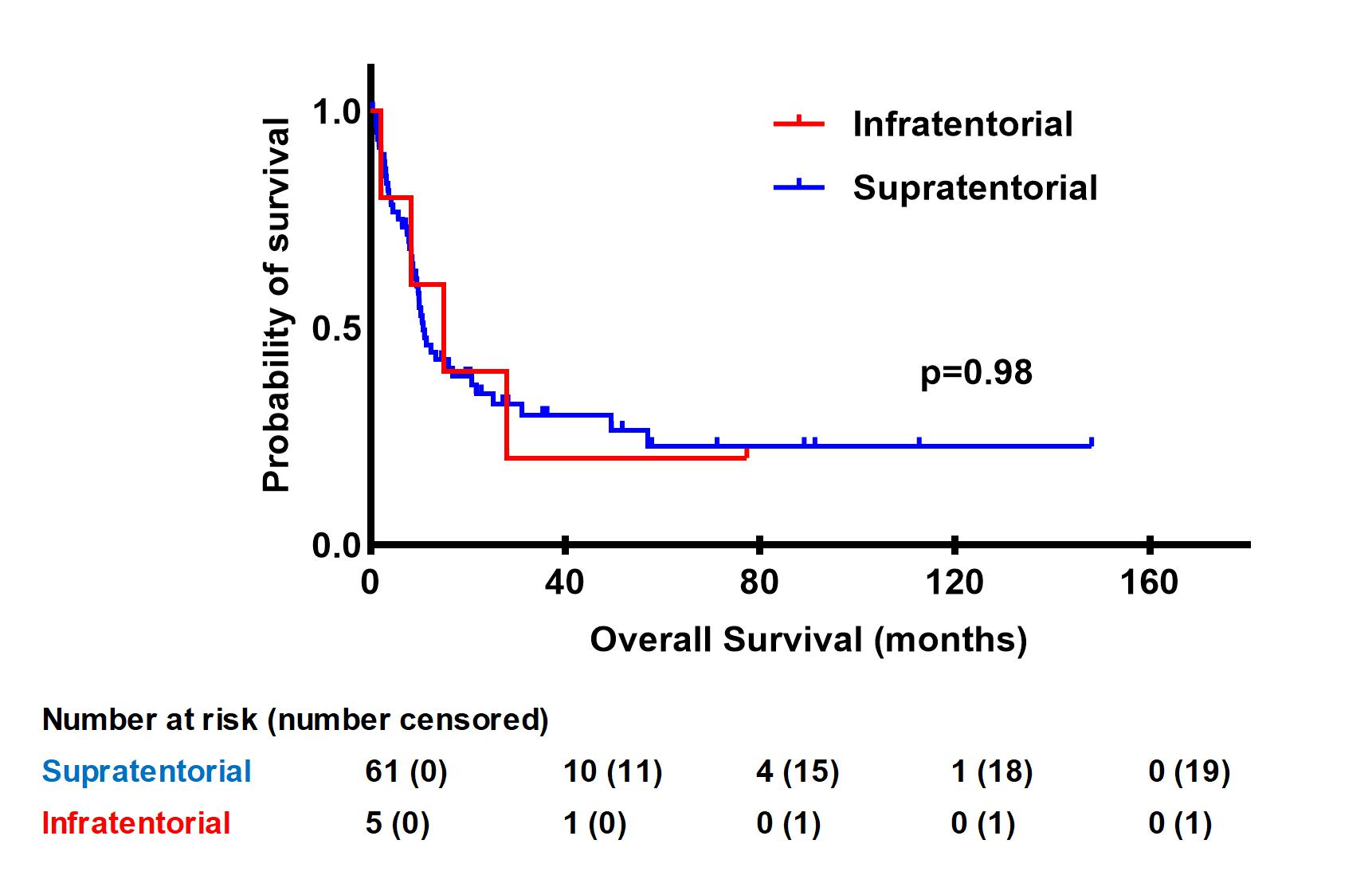

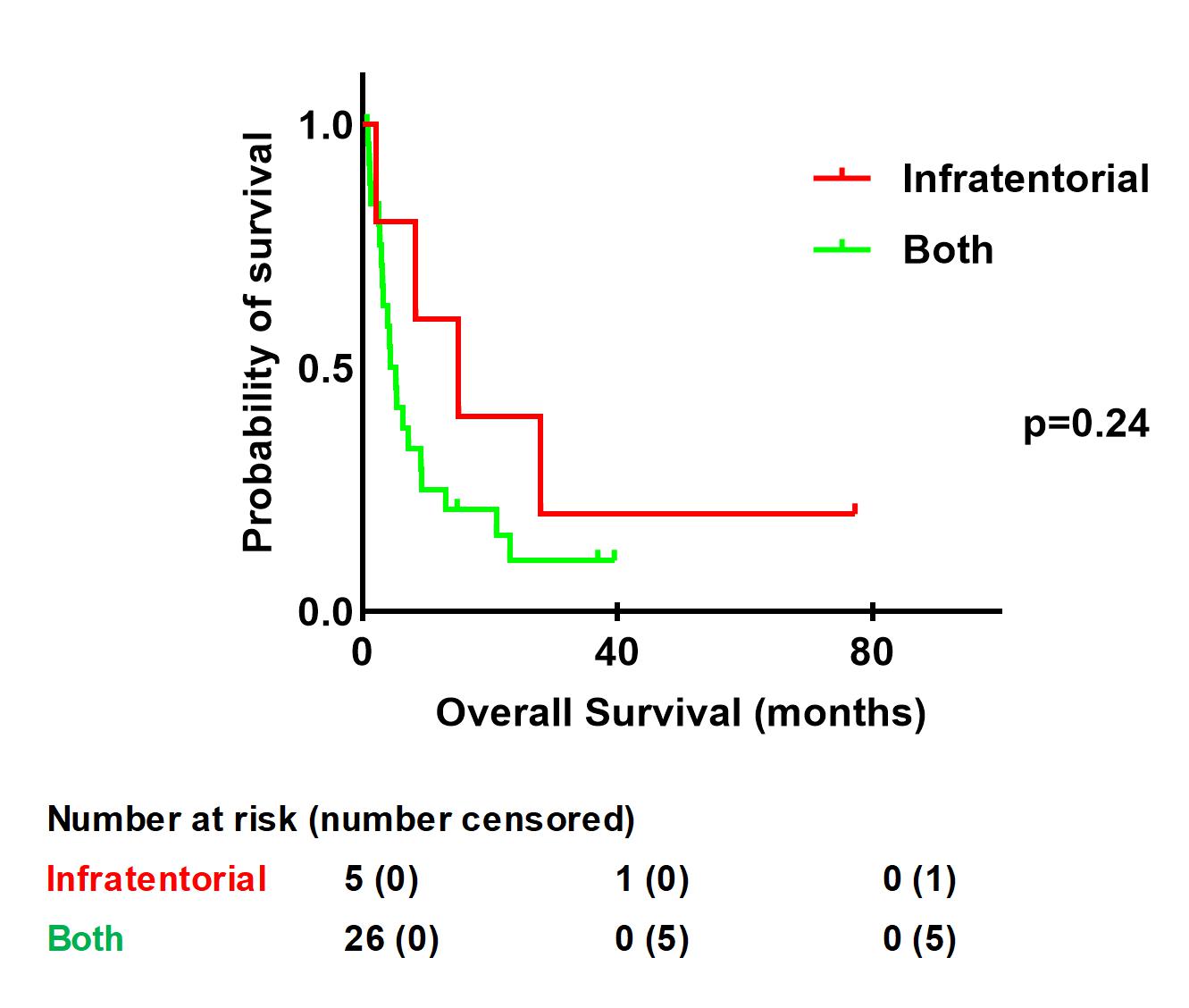
**

Supplemental Figure 4: Kaplan Meier curve for OS in months for supratentorial and infratentorial location groups.

Supplemental Figure 3: Kaplan Meier curve for OS in months for supratentorial and both location groups.

Supplemental Figure 5: Kaplan Meier curve for OS in months for infratentorial and both location groups.
